# Integrative and theoretical research on the architecture of a biological system and its disorder

**DOI:** 10.1007/s12576-019-00667-8

**Published:** 2019-03-13

**Authors:** Shinichi Uchida, Yoshiyuki Asai, Yoshiaki Kariya, Kunichika Tsumoto, Hiroshi Hibino, Masashi Honma, Takeshi Abe, Fumiaki Nin, Yasutaka Kurata, Kazuharu Furutani, Hiroshi Suzuki, Hiroaki Kitano, Ryuji Inoue, Yoshihisa Kurachi

**Affiliations:** 1 Department of Nephrology, Graduate Schools of Medical and Dental Sciences, Tokyo Medical and Dental University, 1-5-45 Yushima, Bunkyo, Tokyo, 113-8519 Japan; 2 Department of Systems Bioinformatics, Yamaguchi University Graduate School of Medicine, Ube, Yamaguchi 755-8505 Japan; 3 Department of Pharmacy, The University of Tokyo Hospital, 7-3-1, Hongo, Bunkyo-ku, Tokyo, 113-8655 Japan; 4 Division of Molecular and Cellular Pharmacology, Department of Pharmacology, Osaka University, Suita, Japan; 5 Center for Advanced Medical Engineering and Informatics, Osaka University, Suita, Japan; 6 Department of Physiology II, Kanazawa Medical University, Ishikawa, 920-0293 Japan; 7 Department of Molecular Physiology, Niigata University School of Medicine, 1-757 Asahimachi-dori, Chuo-ku, Niigata, Niigata 951-8510 Japan; 8 AMED-CREST, AMED, Niigata, Japan; 9 Department of Physiology and Membrane Biology, University of California Davis, Davis, 95616 USA; 10 The Systems Biology Institute, Shinagawa-ku, Tokyo, 108-0071 Japan; 11 Department of Physiology, Fukuoka University School of Medicine, 7-45-1 Nanakuma, Jonan-ku, Fukuoka, 814-0180 Japan

**Keywords:** Systems biology, Physiological experiments, Omics study, Computer simulation, Software platform

## Abstract

An organism stems from assemblies of a variety of cells and proteins. This complex system serves as a unit, and it exhibits highly sophisticated functions in response to exogenous stimuli that change over time. The complete sequencing of the entire human genome has allowed researchers to address the enigmas of life and disease at the gene- or molecular-based level. The consequence of such studies is the rapid accumulation of a multitude of data at multiple levels, ranging from molecules to the whole body, that has necessitated the development of entirely new concepts, tools, and methodologies to analyze and integrate these data. This necessity has given birth to *systems biology*, an advanced theoretical and practical research framework that has totally changed the directions of not only basic life science but also medicine. During the symposium of the 95th Annual Meeting of The Physiological Society of Japan 2018, five researchers reported on their respective studies on systems biology. The topics included reactions of drugs, ion-transport architecture in an epithelial system, multi-omics in renal disease, cardiac electrophysiological systems, and a software platform for computer simulation. In this review article these authors have summarized recent achievements in the field and discuss next-generation studies on health and disease.

**Electronic supplementary material:**

The online version of this article (10.1007/s12576-019-00667-8) contains supplementary material, which is available to authorized users.

## Introduction

The approach of scientists toward the life sciences was dramatically revolutionized by the advent of molecular biology several decades ago and its associated power of ultimate reductionism. This movement was further strongly boosted by the complete sequencing of entire human genome, which is now enabling the enigma of life and disease to be deciphered from the point of view of gene- or molecule-based functionalities. As a consequence, in concert with methodological innovations, entirely new and diverse bodies of knowledge are accumulating at a rapid and unprecedented pace, culminating in deposition of massive amounts of data, such as multiple ‘omics’ and other high-throughput data, in intractably huge databases. However, this flood of information simultaneously necessitates new optimal concepts/logics and tools for analyzing/integrating the data accumulated at multiple functional levels, ranging from molecules to the whole body. The field of systems biology emerged from this serious need for new theoretical/practical frameworks for analyzing and integrating these enormous chunks of information [1–3]. Subsequently, these frameworks have extensively driven many system-level analyses and integrations of existing data, thereby totally changing the directions of medicine, as indicated by the terms ‘precision medicine’ or ‘personalized medicine.’ More recently, the strategic initiatives in most developed countries are aimed at ‘preemptive medicine’ and ‘regenerative medicine,’ in which resilience, repair, and replacement are key concepts. Importantly, the essence of resilience lies in the reversible continuum between health and disease, and thus one of the present aims is to understand how resilience to disease develops and breaks down and to discover the way to prevents this breakdown by appropriate medical interventions and/or regimens aimed at health promotion. This difficult mission will be accomplished not only by discovering the new system-level logics and principles governing normal biological function across all hierarchical levels of the body (from molecules through to organ functions to behaviors/environmental adaptations), but by re-integrating these in such a way as to reconstruct a realistic multi-scale, multi-physics system that can reproduce the continuum of health and disease [4, 5].

This symposium was held during the 95th Annual Meeting of The Physiological Society of Japan (Takamatsu, Japan, on March 28, 2018). It was organized in an eager attempt to explore the newest landscape of modern medicine based on system-level approaches through the presentation of the most recent studies conducted by five front-runners in their respective field of systems biology [6]. The topics of these presentations include: (1) ‘System-based understanding of adverse reactions of molecularly targeted drugs’ by Yoshiaki Kariya, Masashi Honma, and Hiroshi Suzuki; (2) ‘Multi-omics approaches to chronic kidney disease’ by Shinichi Uchida; (3) ‘Physiological architecture of the ion transport system in the epithelial tissue of the inner ear’ by Hiroshi Hibino, Fumiaki Nin, and Yoshihisa Kurachi; (4) ‘Robustness and vulnerability in cardiac electrophysiological systems: from a viewpoint of dynamical system theory’ by Kunichika Tsumoto, Yasutaka Kurata, Kazuharu Furutani, and Yoshihisa Kurachi; and (5) ‘Multilevel systems medicine and software platform’ by Yoshiyuki Asai, Takeshi Abe, and Hiroaki Kitano. Detailed synopses of these presentations are presented in the following sections of this review.

## System-based understanding of adverse reactions of molecularly targeted drugs (Yoshiaki Kariya, Masashi Honma, and Hiroshi Suzuki)

Here we present an approach used to understand adverse reactions associated with molecularly targeted drugs. During the last two decades, several molecularly targeted drugs for cancer treatment have been developed, primarily based on recent advances in cancer cell biology. One such example is the identification of mutations in the epidermal growth factor receptor (EGFR) gene in lung cancer cells [7]. It has been reported that such EGFR mutations enhance cell growth and that inhibiting the mutated EGFR gene suppresses this enhanced cell growth. This has led to the development and clinical use of several EGFR inhibitors, such as gefitinib, erlotinib, afatinib, and osimertinib. In addition to EGFR inhibitors, tyrosine kinase inhibitors (TKIs), a class of molecules used very extensively in the clinical setting, are designed to inhibit the activity of target kinases by blocking their ATP binding pocket in the kinase domain. As the pocket structure is similar among various kinases, the kinase inhibitors often inhibit the activity of kinases that are unintended targets—i.e., “off-target” kinase. The off-target kinases are reported to be associated with adverse drug reactions (ADRs), which are rarely observed with chemotherapy using cytotoxic anti-tumor drugs; therefore, the prediction and prevention of these ADRs are clinically important. However, although the mechanisms underlying the anti-tumor effect of molecularly targeted drugs have been clarified, the mechanism of ADRs is obscure as it is difficult to identify the kinases, including the off-target kinases, associated with the ADRs. In other words, numerous kinases can be a mediator of ADRs in terms of drug–kinase binding, which makes it difficult to clarify the mechanism analysis of ADRs. Systems biology can facilitate an understanding of these physiological responses as an integrative outcome of numerous complicated components. In this regard, an approach based on systems biology may be compatible with the mechanism analysis of ADRs and, therefore, we analyzed two kinase inhibitors.

The first analysis is about severe skin rash associated with erlotinib [8]. The occurrence rate of skin rash due to erlotinib, an EGFR inhibitor used clinically to treat various cancers, is extremely high (50%) compared with that due to gefitinib, another EGFR inhibitor (10%). Skin rash has on occasion forced treatment discontinuation, and it is a clinical problem to be overcome. To elucidate the mechanism, we narrowed down the candidate kinases using the comprehensive data of affinities between kinase inhibitors and kinases reported previously [9]. We observed that the dissociation constant (Kd) value of EGFR is tenfold lower than that of most kinases, suggesting that erlotinib is a selective EGFR inhibitor. However, to elucidate the pharmacological phenomenon, it is not sufficient to know the biochemical properties as drug concentration should also be considered in order to identify those kinases affected by clinical conditions. Therefore, we compared the kinase occupancies by erlotinib with those by gefitinib at their clinical concentrations. Two kinases—STK-10 and SLK—were found to be erlotinib-specific targets. As these two kinases have been reported to be functional in immune responses, especially in the suppression of interleukin-2 (IL-2) secretion in T cells, the effect of STK-10 and SLK knockdown in the presence or absence of drugs was tested. In the control knockdown condition, supplementation of erlotinib at clinical concentration enhanced IL-2 secretion in Jurkat cells, whereas supplementation of gefitinib did not. Additionally, the knockdown of SKT-10 enhanced IL-2 secretion, whereas the knockdown of SLK did not. Furthermore, there was no additive enhancement of IL-2 secretion by STK-10 knockdown together with erlotinib supplementation. These observations suggest that SKT-10 is a mediator of enhanced IL-2 secretion with erlotinib supplementation. IL-2 is a proinflammatory cytokine and its enhanced secretion may be a putative mechanism for severe skin rash. To confirm this hypothesis in vivo, we performed an irritant hypersensitivity assay using ddY mice, measuring earflap swelling after topical application of croton oil as an indicator of irritant hypersensitivity. When erlotinib or gefitinib was administered to the mice at the dosage presenting an area under the curve and peak concentrations comparable to human clinical cases, aggravation of skin inflammation was observed only in the erlotinib-administered group. Additionally, following the administration of an anti-IL-2 neutralizing antibody, the enhanced inflammation by erlotinib was significantly reduced. These in vivo observations suggest that severe skin rash observed in clinical situations is driven by enhanced IL-2 secretion via STK-10 “off-target” inhibition. Therefore, if we assume that the anti-tumor effect of erlotinib is mostly mediated by EGFR inhibition, it is theoretically possible to reduce the risk of skin rash with a negligible loss in therapeutic effect by optimizing the dose to inhibit EGFR but not STK-10. Collectively, the integration of comprehensive kinase affinity data and pharmacokinetic property is a powerful approach to elucidate the mechanism of TKI-associated ADRs.

**Fig. 1:**
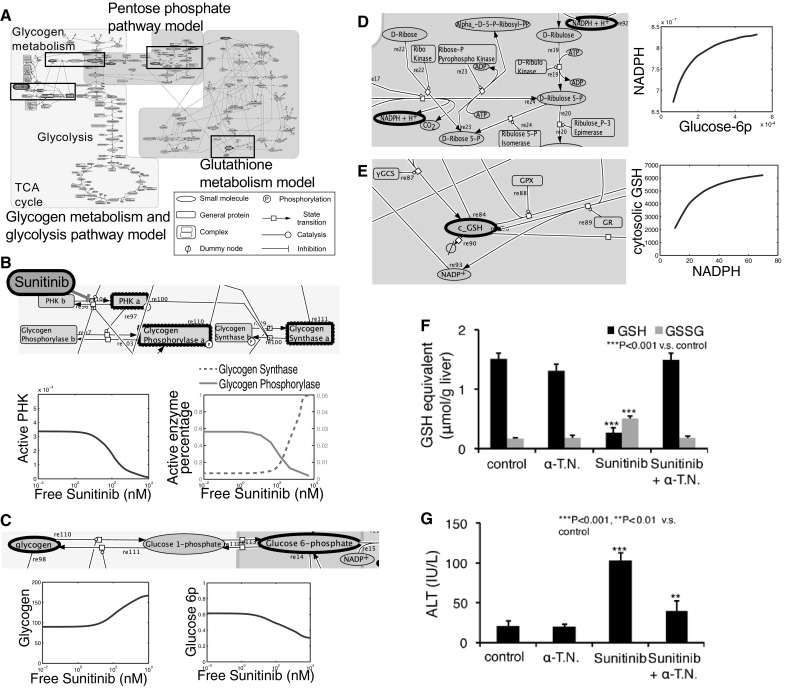
Effect of sunitinib on metabolic pathways were simulated by sequential simulation of previously reported models. **a–e** The simulation result suggests that sunitinib treatment induces oxidative stress. Phosphorylase kinase (*PHK*) gamma-related pathways were integrated and simulated using three divided models (**a**) (for expanded version, see Electronic Supplementary Material figure). The pathways highlighted by black-boxed regions in **a** are enlarged in **b–e**, which also display the simulated change in the amount of different molecules as a function of free sunitinib concentration. **f**, **g** The prediction was confirmed in in vivo experiments using a mouse model. A decrease in the glutathione/oxidized glutathione (*GSH*/*GSSG*) ratio was observed in many organs of mice treated with sunitinib. In addition, elimination of oxidative stress by α-tocopherol nicotinate (*α-T.N.*) can prevent organ toxicities.* ALT* Alanine aminotransferase,* GPx* glutathione peroxidase,* GR* glutathione reductase. The results of liver toxicity analyses are shown as representative data

**Fig. 2:**
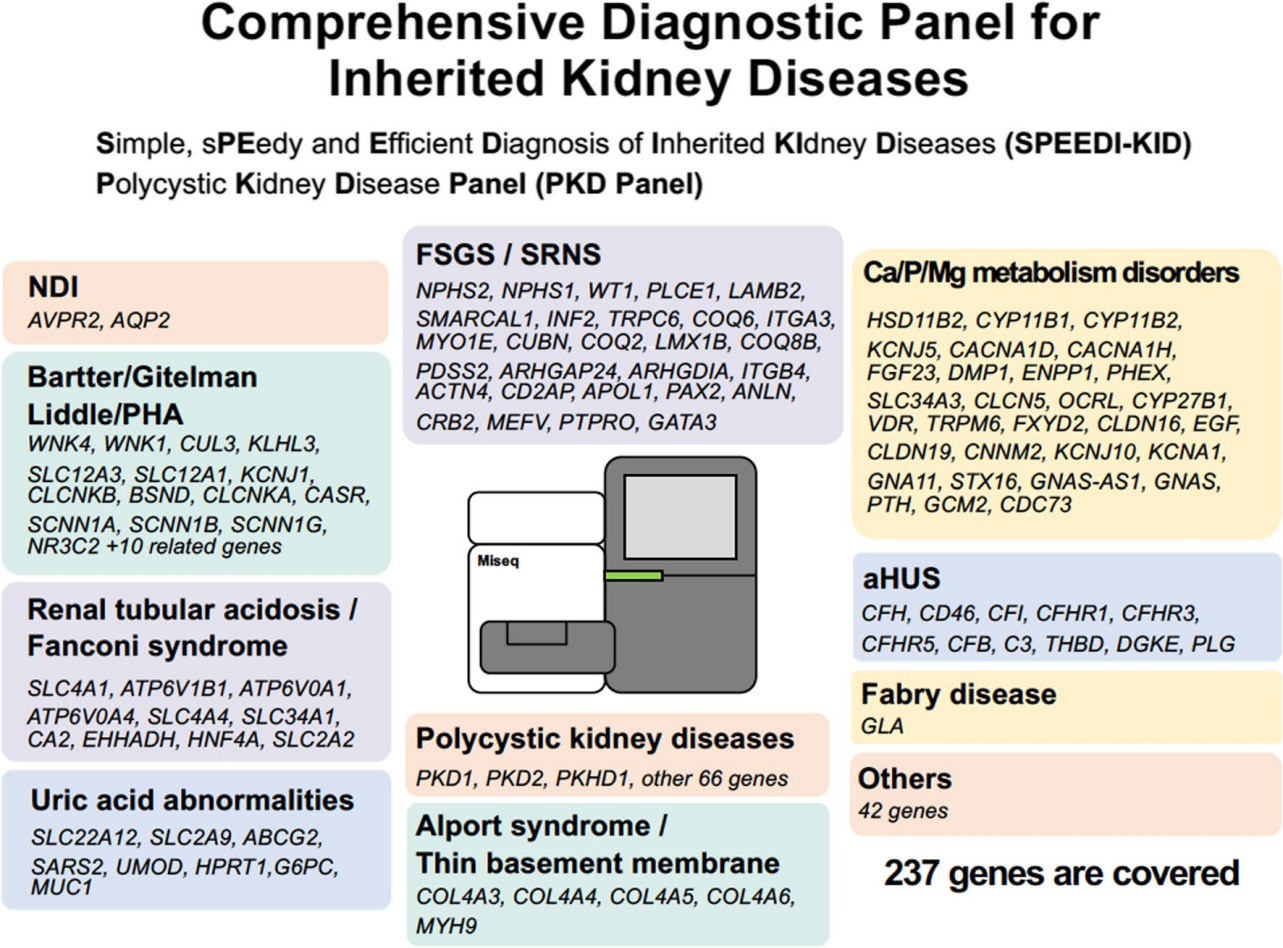
Targeted genes covered in the next-generation sequencing (NGS) panel. A total of 237 genes known to be responsible for various inherited kidney diseases are included. *aHUS* Atypical hemolytic uremic syndrome, *FSGS* focal segmental glomerulosclerosis, *NDI* nephrogenic diabetes insipidus, *PHA* pseudohypoaldosteronism, *SRNS* steroid resistant nephrotic syndrome

The second analysis is about sunitinib-related ADRs [10]. Sunitinib is a multiple kinase inhibitor that is used clinically to treat various cancers. It has been reported that treatment with sunitinib causes ADRs, including platelet depletion, hypothyroidism, and cardiac and hepatic toxicities. Moreover, the occurrence rate of these ADRs is higher in patients treated with sunitinib than in patients treated with sorafenib, another multiple kinase inhibitor. As the occurrence of ADRs is one of major reasons for dose reduction, preventing/overcoming these ADRs is a clinically important objective, we performed a mechanism analysis of sunitinib-related ADRs. Similar to the mechanism analysis of erlotinib, we compared the kinases occupied by sunitinib and sorafenib. Phosphorylase kinase gamma subunit 1/2 (PHKG1/2), an enzyme that catabolizes glycogen decomposition to glucose-1-phosphate, was found to be inhibited selectively by sunitinib at its clinical drug concentration. However, we were unable to identify the mechanism of ADRs only by this enzymatic property. Therefore, we attempted to integrate the molecular network surrounding this enzymatic reaction and subsequently found that the PHKG-related reactions were linked to the glycolysis/gluconeogenesis, pentose phosphate, and glutathione redox pathways (Fig. 1a). To narrow down the possible mechanism of ADRs, we also attempted to simulate these pathways in the presence of sunitinib. However, there are no reported quantitative dynamic simulation models that encompass all of these pathways, although there are several models that include some of these pathways. As each model was optimized to the background in which the model was constructed, it is difficult to build one large model by simply combining several models. Instead, we employed a sequential simulation approach for several parts of the models and made the following semi-quantitative predictions (Fig. 1b): (1) sunitinib treatment lowers glucose-6-phosphate synthesis in the glycolysis pathway (Fig. 1c); (2) lowered glucose-6-phosphate synthesis decreases the NADPH/NADP^+^ ratio (Fig. 1d); and (3) a lowered NADPH/NADP^+^ ratio decreases the cytosolic concentration of reduced form of glutathione (GSH) (Fig. 1e). As cytosolic GSH protects intracellular components from oxidative stress, the simulation resulted in the hypothesis that sunitinib enhances oxidative stress, thereby damaging various organs. To validate this hypothesis, we measured several metabolites and oxidative stress markers in various organs using C57BL6/J mice dosed with sunitinib or sorafenib mixed in feed (Fig. 1f, g). The sunitinib-administered mice exhibited an exacerbated organ function marker, a decreased GSH to oxidized glutathione (GSSG) ratio, and an increased level of the oxidative stress marker TBARS in all tested organs (liver, heart, and thyroid) and platelets, whereas the sorafenib-administered mice did not exhibit such disruptions. Furthermore, the concomitant administration of an anti-oxidant protected against exacerbation of organ function. These results indicate that the mechanism of sunitinib ADRs is due to excess oxidative stress caused by perturbation of the metabolic pathway via PHKG1/2 inhibition. We also confirmed that the concomitant administration of sunitinib and alpha-tocopherol nicotinate, an antioxidant, did not affect the antitumor effect of single treatment of sunitinib in in vivo experiments using xenograft mice, further indicating that system-based simulation and prediction is an effective strategy for mechanism analysis of TKI-related ADRs.

For future application of our approaches, we should consider the limitations to be overcome. One of the key factors affecting the success of sunitinib-related ADR analysis is the properties of the metabolic pathway models used. As metabolic pathway models are well constructed and based on detailed knowledge of actual metabolic network structures, there are few undescribed and unknown pathways largely affecting simulation results. Additionally, metabolite concentrations and related enzymatic activity are directly measurable; therefore, kinetic parameters of each model component can be determined in a relatively accurate manner. Thus, simulation based on the previously established models helps to predict metabolic events other than the events focused on in the original analysis. To predict ADRs, there are also situations in which we would like to simulate signal transduction-related events, such as cell death, in addition to metabolic events. However, signal transduction models are established in a relatively less reductionistic manner due to experimental restrictions; therefore, there are few models widely available to analyze cell types other than the originally cells focused upon in the original studies. In many cases, to establish the kinetic models describing the intracellular singling pathways, it is necessary to fine-tune specific cell types to reproduce experimental observations. Therefore, the parameter values used in a particular model cannot be easily transferred to another model describing other cell types. The comprehensive parameter determination approach might be helpful to overcome this problem. In the analysis of physiology-based pharmacokinetic models, trials to identify a large number of parameter combinations to reproduce the observed drug concentration curve have been carried out [11]. Based on analysis of obtained parameter combinations, it was possible to calculate the representative parameter values and their respective variability in complicated models. If we can obtain these variabilities together with the parameter values in the signal transduction models using just such an approach, the transferability of parameter values between different cell types will be improved as the variabilities may include information on differences in cell types. This information will greatly advance and expand the availability of simulation models. To conclude, although many tasks remain regarding the availability of simulation models, we show here that system-based analyses, including both comprehensive data analysis and model simulations, are useful for analyzing and predicting pharmacological outputs, including ADRs.

## Multi-omics approaches to chronic kidney disease (Shinichi Uchida)

Chronic kidney disease (CKD) is a major global health problem, and in Japan it is estimated that about 13% of the adult population have CKD. The prevalence of end stage kidney disease (ESKD) is also rapidly increasing. Renal replacement treatment in expensive. It was reported that about 40,000 patients were newly introduced to renal replacement therapy in Japan in a 1-year period, resulting in more than 300,000 patients being currently on dialysis in Japan. CKD is also a well-known risk factor for cardiovascular mortality and morbidity. Thus, early recognition and treatment of CKD are important to prevent progression to cardiovascular diseases and ESKD. However, drugs specific for the treatment of CKD are still lacking because there is insufficient knowledge on the mechanism of how the CKD kidney continues to fail irrespective of the primary cause. To identify novel target molecules and mechanisms to develop drugs for CKD, our group conducted multi-omics approaches to CKD. The methods used in mouse CKD models were transcriptomics using microarrays and whole transcriptome shotgun sequencing (RNA-Seq) by next-generation sequencing (NGS), epigenomics, and metabolomics, including lipidomics. The CKD model we used was a mouse 5/6 nephrectomy model in C57BL/6 and 129/SvJ mice, as CKD-resistant and -prone strains, respectively. Previous quantitative trait locus (QTL) analyses and single nucleotide polymorphism (SNP) data in both strains were also considered with these omics data. We also conducted human genomics focusing on familial CKD patients whose etiology of CKD was unknown. For this purpose, we prepared a comprehensive diagnostic panel for kidney diseases that simultaneously provided us with all of the exon information of the approximately  200 genes related to kidney diseases through NGS. This approach resulted in the identification of several candidate genes which could be important in the progression of CKD in the mouse CKD models and in human genetics; validation studies are currently under way. The utility of and the problems encountered in omics studies and future directions in system biology are discussed in this section.

Omics analyses imply a comprehensive assessment of a set of molecules, and studies using omics data have been driven by technological advances that have made cost-efficient and high-throughput analyses of biological molecules possible. In the branch of molecular biology known today as genomics, expression arrays were first generated in the late 1990s, leading to analyses of QTLs and the genome-wide association studies (GWAS). Among the various “omics”, genomics has been particularly successful because (1) a high-quality reference of the human genome has been established, (2) high-throughput genotyping is possible by NGS, and (3) large coordinated cohorts are available and cohort studies are being conducted. However, additional approaches for elucidating the causes of human diseases are still needed since (1) only a fraction of Mendelian diseases have been identified, and (2) the same genetic variants, even in the same family, often cause different phenotypes, probably due to differences in environmental and genetic backgrounds. Each type of omics data other than genomics data just provides a list of differences associated with diseases. Analysis of only one omics data type only shows correlations, mostly reflecting reactive processes rather than causative ones. Therefore, integration of different omics data types is often necessary to elucidate potential causative changes that lead to diseases. In this respect, the development of system biology technologies that make possible the integration of different omics data and the identification of true causative changes is urgently needed.

CKD is a common complex disease entity that develops over time and is influenced by both genetic and environmental factors. Therefore, to clarify a final common mechanism of CKD progression irrespective of primary kidney diseases, coordinated sets of several layers of omics data are necessary, even at multiple time points during the progression of the disease, from multiple disease-relevant tissues. To fulfill this criterion, researchers have to consider both animal and human studies.

In human studies, genomics has been one of the most powerful tools for identifying causative genes for diseases. GWAS have been performed, and the results from more than one study have suggested that SNPs in the* UMOD* gene encoding uromodulin are associated with CKD progression [12, 13]. Uromodulin, also called Tamm–Horsfall glycoprotein, is abundantly produced in the thick ascending limb of Henle’s loop in the kidney and excreted in the urine. The physiological role of this protein has not been fully established since knockout mice do not show any apparent phenotypes. In addition to the association of* UMOD* expression with CKD progression identified in GWAS,* UMOD* has also been identified as a gene responsible for Mendelian forms of kidney diseases, such as familial juvenile hyperuricemic nephropathy (FJHN) and medullary cystic kidney diseases (MCKD). We recently identified * UMOD* mutations in three familial CKD pedigrees; their clinical phenotypes are not those of FJHN and MCKD, and they are believed to be CKD of unknown etiology. It is well known that CKD phenotypes, even those caused by the same mutation, vary significantly among patients. Based on these observations, we speculated that powerful genes such as* UMOD* causing the Mendelian form of kidney diseases might be involved in more common mechanisms of CKD progression as modifier genes. Increased or decreased expression of SNP(s) or partial loss-of-function mutations may not induce clear phenotypes, but either may be present more commonly in chronically progressive diseases such as CKD as modifier genes. In this context, the identification of mutations of previously identified causative genes for common kidney diseases in CKD patients is essential. Therefore, we built a comprehensive diagnostic panel for kidney diseases which tells us simultaneously all of the exon information of the approximately  200 genes related to kidney diseases by NGS [14] (Fig. 2). Further analyses of CKD patients with unknown disease etiology using this panel would clarify new therapeutic targets of CKD other than* UMOD*.

We also applied multi-omics approaches in experiments using mouse CKD models. Animal studies have certain advantages over human studies, including better reproducibility, better assessment of accurate phenotypes, increased ease in obtaining biological samples, and higher constancy of environmental factors. C57BL/6 and 129/SvJ mice are CKD-resistant and CKD-prone strains, respectively. We attempted to determine the source of this difference between the two strains using transcriptome, proteome, and metabolome data combined with SNP data and results from previous QTL analyses on both strains. Among the omics performed, we felt that metabolomics might be a powerful tool for CKD studies since (1) the kidney is the major organ involved in the excretion of various metabolites and (2) the levels of various metabolites in the body are dramatically affected in CKD. There may be metabolites that are primarily responsible for CKD progression, either by affecting enzymatic activity, gene transcription, and/or protein translation and modification. Something important for CKD progression not identified by other omics data can be found in metabolomics.

In summary, the omics approach is indispensable when the aim is to identify something new in biological studies. Methods to integrate multi-omics data to find the true causative element are not standardized. Consequently, there needs to be a concerted effort to obtain more coordinated sets of omics data of a certain quality.

## Physiological architecture of the ion transport system in the epithelial tissue of the inner ear (Hiroshi Hibino, Fumiaki Nin, and Yoshihisa Kurachi)

**Fig. 3:**
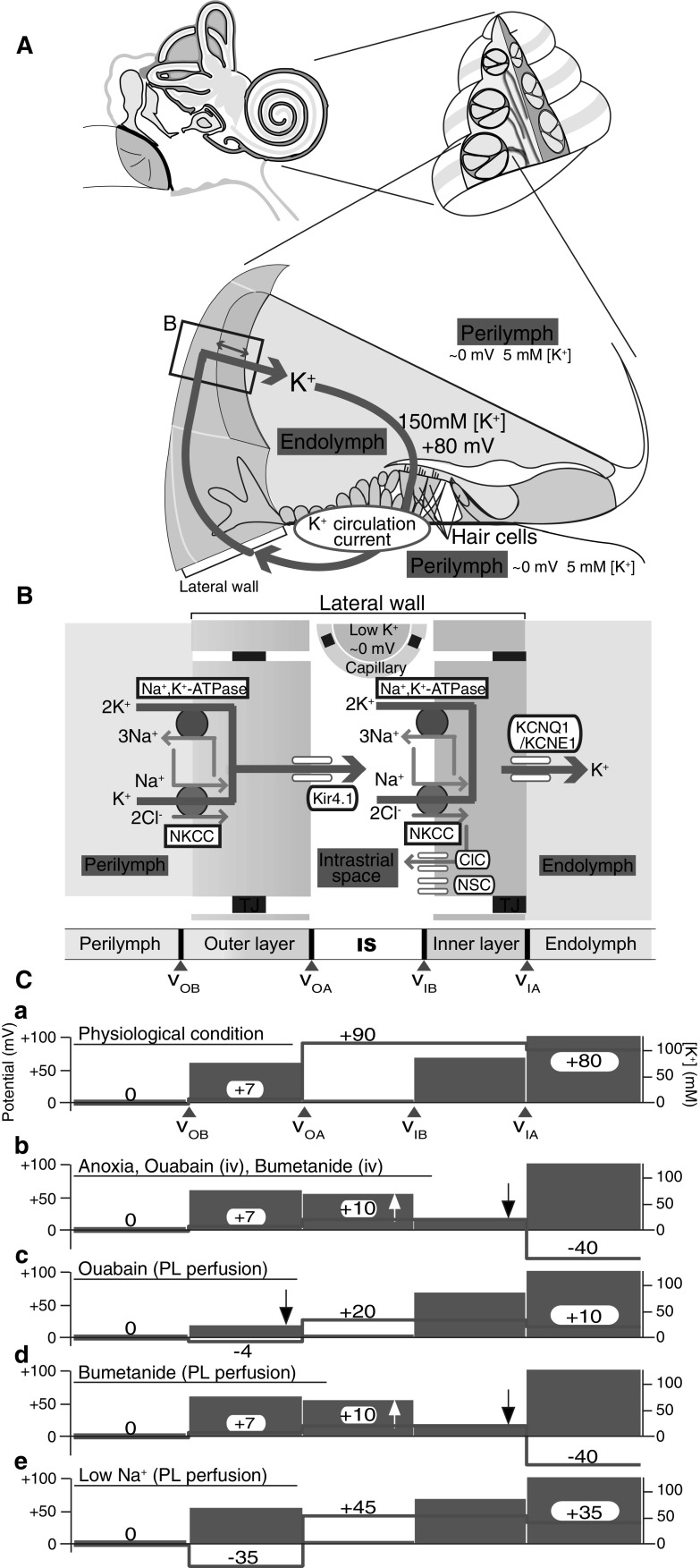
Electrochemical properties of the mammalian cochlea. **a** Structure of the cochlea. Overall view of the human ear is illustrated in the upper left panel. The upper right panel shows a cross-section of the cochlea. In the lower panel, the tissue and cellular compositions of the cochlea as well as the electrochemical profiles of endolymph and perilymph (*PL*) are shown. Note that potassium ions (K^+^) unidirectionally circulate between the two types of lymph (endolymph and perilymph) through the lateral cochlear wall (described as a K^+^ circulation current). The boxed area in **a** is enlarged in **b**. **b** Structure and molecular architecture of the lateral wall. The scheme shows that this tissue is made up of outer and inner layers; between the two layers lies the intrastrial space (*IS*). Ion channels and transporters, which are likely to play a crucial role in the maintenance of endolymphatic high potential and of the K^+^ circulation current, are also described.* ClC* ClC-K/barttin-type chloride ion (Cl^−^) channel,* Kir4.1*,* KCNQ1/KCNE1* K^+^ channels,* NKCC* Na^+^, K^+^, 2Cl^−^-cotransporter,* NSC* nonselective cation channel. *v*_*OB*_, *v*_*OA*_, *v*_*IB*_, *v*_*IA*_ Membrane potential on the basolateral (subscript B) and apical (subscript A) surfaces of the outer (subscript O) and inner layer (subscript I). **c** Potential and ion profiles of the lateral wall, perilymph, and endolymph. Red lines and numbers and blue bars indicate the potential relative to perilymph and K^+^ concentration [K^+^] , respectively. In each of the five panels (**a**–**e**), representative in vivo measurements obtained under the different conditions described above the blue bars are shown. Ouabain and bumetanide are inhibitors of sodium ion (Na^+^), K^+^-ATPases and NKCC, respectively. The upward and downward arrows show the increase and decrease of [K^+^], respectively. *iv* Intravenous injection. Images in **a**–**c** were reproduced and adapted from Nin et al. [24]

In this section we discuss an ion transport system that maintains and regulates the electrochemical milieu in the cochlea of the mammalian inner ear. This tiny organ harbors two different extracellular solutions, namely, perilymph and endolymph. Perilymph has an ionic composition similar to that of blood plasma, while endolymph contains a high potassium ion concentration ([K^+^]) of 150 mM and has a highly positive endocochlear potential (EP) of + 80 mV relative to perilymph (Fig. 3a) [15, 16]. The apical membranes of sensory hair cells, which express mechanoelectrical transduction (MET) channels, are exposed to endolymph, and their basolateral surfaces are bathed in perilymph. In response to acoustic stimuli, K^+^ from endolymph enters into the hair cells through the MET channels [17]. This process depolarizes the hair cells and induces the release of a neurotransmitter from these cells that triggers propagation of the signals to the brain. The highly positive EP increases the driving force for the K^+^ entry and therefore contributes to the high sensitivity of hair cells.

It seems probable that in the cochlea, K^+^ unidirectionally flows from endolymph to perilymph through the hair cell layer and then returns to endolymph across the lateral cochlear wall, an epithelial-like tissue (Fig. 3a) [18, 19]. This K^+^ cycling or K^+^ circulation current may constantly occur even without acoustic stimulation because under such conditions a subpopulation of MET channels are opened [20, 21]. The K^+^ circulation current is likely to be driven by K^+^ transport machineries in the lateral wall. The lateral wall is thought to consist of two layers: an outer and inner layer, respectively [19, 22]. In each layer, the apical surface expresses K^+^ channels whereas the basolateral surface bears two different K^+^ uptake transporters, Na^+^, K^+^-ATPases and the Na^+^, K^+^,2Cl^−^-cotransporter (NKCC) (Fig. 3b) [23, 24]. It has been proposed that these K^+^ transport proteins are involved in the unidirectional transport of K^+^. Moreover, physiological experiments point to the key roles of the channels and transporters in formation of the EP. Notably, a few research groups have analyzed the extracellular space between the inner and outer layers of the lateral wall, called the intrastrial space (IS), with a K^+^-selective microelectrode that can simultaneously measure [K^+^] and potential. The authors of these studies reported that the IS has a low [K^+^] of several millimoles per liter and a highly positive potential similar to the EP (Fig. 3b, c) [22, 25]. There is a large [K^+^] gradient across the outer layer’s apical membrane that is dominated by K^+^ channels (Fig. 3c). Based on these results, it is hypothesized that the positive IS potential is composed primarily of a K^+^ diffusion potential (namely, the K^+^ equilibrium potential, E_K_) and a major origin of the EP [22, 26, 27]. Recently, our in vivo measurements with K^+^-selective microelectrodes not only proved this hypothesis but also identified an additional element critically involved in the generation of the EP [28]. Imposition of anoxia, which inhibits mainly K^+^ uptake transporters on the inner layer’s basolateral surface, in animals augments [K^+^] in the IS with a minimal change in [K^+^] inside the outer layer, thus reducing the amplitude of the K^+^ diffusion potential and thereby also the IS potential (Fig. 3c). Simultaneously, the perturbation decreases the [K^+^] inside the inner layer and negligibly affects the [K^+^] in endolymph. This situation increases the K^+^ diffusion potential on the inner layer’s apical surface, which expresses abundant K^+^ channels. Owing to the emergence of this potential difference, the EP reaches a negative value. Accordingly, the EP depends primarily on two K^+^ diffusion potentials in the lateral wall: the one on the outer layer’s apical surface and the other on the inner layer’s apical surface (Fig. 3c) [24, 28].

**Fig. 4:**
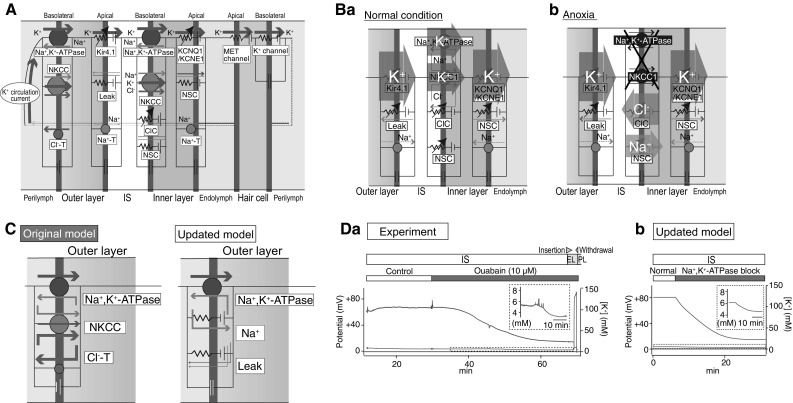
In silico reproduction of electrochemical dynamics in the cochlea. **A** An outline of the original Nin–Hibino–Kurachi (*NHK*) model [29]. This model is characterized by outer and inner layers in the lateral wall and the hair cell layer, respectively. The profile of each of the six surfaces is represented by electrical circuits that contain ion channels and transporters. These circuits are connected in series and drive the K^+^ circulation current under normal conditions (see Fig. 3a). *ClC* ClC-K/barttin-type Cl^−^ channel, *Cl*^*−*^*-T* Cl^−^-transporter, *MET* mechanoelectrical transduction, *Na*^*+*^*-T* Na^+^-transporter. Reproduced and adapted from Nin et al. [24]. **B** Ionic dynamics in the lateral wall. **Ba** and **Bb** indicate major ionic flows underlying the maintenance of or change in the electrochemical properties of the cochlea under normal and anoxic conditions. See the main text for details. Reproduced and adapted from Nin et al. [24]. **C** The updated NHK model. In the recent model (right panel), a few ion transport molecules incorporated into the basolateral surface of the outer layer were changed on the basis of the experimental observations, as described in the main text and in references [33, 35]. Left panel indicates the corresponding portion in the original model. Reproduced and adapted from Yoshida et al. [36]. **D** Comparison of the experimental and simulated results. **Da** A microelectrode sensitive to the potential (red) and [K^+^] (blue) was inserted from PL into endolymph (*EL*) in a live guinea pig. After the passage, while the electrode was held in the IS, control artificial perilymph or a solution containing ouabain (10 µM), a blocker of Na^+^, K^+^-ATPases, was perfused into the perilymphatic space. The [K^+^] measurement highlighted by the dashed box is expanded in the inset. A wedge above the trace shows the period when the electrode was moved forward or backward. **Db** Illustration of a simulation of the experimental conditions shown in **a** via the recently updated NHK model. [K^+^] behavior indicated by the dashed box is enlarged in the inset. Reproduced and adapted from Yoshida et al. [35]

Despite these observations, the relevance of the K^+^ circulation current throughout the cochlea to the EP remains uncertain because this current and the underlying ionic currents via the channels and transporters are unmeasurable in vivo. To address these issues, we have employed an in silico approach [29]. In our electrochemical model, K^+^ circulates across six membrane domains, i.e., the apical and basolateral surfaces of the two layers in the lateral wall and those in the hair cell layer (Fig. 4a). Each surface is profiled by Hodgkin–Huxley-type equations that represent the function of the channels and transporters. Note that the model includes Cl^−^ channels and nonselective cation channels experimentally identified on the inner layer’s basolateral surface [30, 31]. The electrical circuits on these six surfaces are connected in series to drive the K^+^ circulation current throughout the cochlea. Owing to this arrangement, the circulation current stems from the sum of the ionic currents via all of the ion transport proteins on each surface and can control membrane potentials and ionic concentrations in the extracellular and intracellular spaces of the cochlea. This Nin–Hibino–Kurachi (NHK) model replicates the majority of experimental data on the electrochemical properties of endolymph and of the lateral wall under different conditions [29].

The model can theoretically explain the mechanism underlying alterations in the lateral-wall [K^+^] profile during anoxia that may represent ischemic conditions in the cochlea (Fig. 4b) [24, 29]. Under normal conditions, the circulation current is set to be composed purely of K^+^. When the model is configured for anoxic conditions, where K^+^ uptake transporters on the inner layer’s basolateral surface are inhibited, a fraction of the circulation current across this surface switches from K^+^ to other ions via Cl^−^ channels and nonselective cation channels. As a consequence, K^+^ inflow exceeds K^+^ outflow in the IS and vice versa in the inner layer, causing [K^+^] in the IS to increase and [K^+^] inside the inner layer to decrease, as observed in the experiments (see Fig. 3c). These changes in the [K^+^] profile result in a reduction of the IS potential and EP. Accordingly, the circulation current is likely to be coupled to the EP.

The ion transport machineries on the basolateral and apical surfaces of the inner layer and on the apical surface of the outer layer have been actively characterized by in vivo and in silico assays, as described above. Nevertheless, the basolateral surface of the outer layer has not yet been sufficiently analyzed. This surface expresses the proteins Na^+^, K^+^-ATPases and NKCC and manifests a resting membrane potential (RMP) of + 5 to + 12 mV in vivo (Fig. 3c) [28, 32, 33]. In particular, because of this unusually positive value of RMP, the K^+^ diffusion potential across the apical surface of the outer layer can contribute to the formation of the highly positive values of the IS potential and EP (Fig. 3c). The K^+^ uptake transporters and unique RMP were recently assessed in our electrophysiological experiments in live animals. First, perfusion into the perilymph—to which the outer layer’s basolateral surface is exposed—of a blocker for Na^+^, K^+^-ATPases reduces the [K^+^] inside the outer layer, whereas perfusion of an inhibitor of NKCC affects this [K^+^] property only negligibly (Fig. 3c) [32, 34]. Therefore, Na^+^, K^+^-ATPases—but not NKCC—convey K^+^ in vivo. The second observation was obtained with perilymphatic infusion of solutions that have a different ionic composition (Fig. 3c); the results indicate that the outer layer’s basolateral surface is more permeable to Na^+^ than to K^+^ and Cl^−^ under physiological conditions, and this unusual property critically contributes to the positive RMP [35].

On the basis of these findings, the NHK model has been updated (Fig. 4c) [36]. In this model, the basolateral surface of the outer layer harbors only Na^+^, K^+^-ATPases, Na^+^ conductance, and leak conductance, and lacks NKCC. These three machineries are assumed to be functionally coupled together to locally recycle Na^+^; on the basolateral surface, this arrangement allows Na^+^, K^+^-ATPases to unidirectionally mediate K^+^ transport (which constitutes the circulation current) and to maintain the positive RMP. With appropriate parameter settings, the model approximately reproduces experimental measurements in various extracellular and intracellular spaces of the lateral wall and in endolymph under normal conditions. Moreover, pharmacological inhibition of the outer-layer Na^+^, K^+^-ATPases is a condition that is inaccessible to the original NHK model but which can be simulated with the updated model. In vivo, perilymphatic perfusion of such an inhibitor reduces [K^+^] inside the outer layer as well as [K^+^] in the IS (Fig. 3c). Because the former changes more strongly than the latter, the [K^+^] gradient across the apical surface of the outer layer is diminished, decreasing the IS potential and the EP. All of these measurements are reasonably reproduced in the updated model. These observations reinforce the validity of the assumption in the model. It should be emphasized that although the outer layer’s basolateral surface expressing Na^+^, K^+^-ATPases does not face the IS, its [K^+^], which is controlled by the circulation current flowing throughout the cochlea, significantly changes (Fig. 3d). This result implies that Na^+^, K^+^-ATPases, Na^+^ conductance, and leak conductance, which are coupled on the outer layer’s basolateral surface, are indeed linked to the circulation current as assumed in the model.

As described above, computer simulations and various types of experiments conducted by our laboratory and/or other groups have unveiled most of the physiological architecture necessary for the formation of the EP and circulation current. Nevertheless, several critical components remain to be determined. One example is a functional protein responsible for high Na^+^ permeability on the outer layer’s basolateral surface that exhibits the unique RMP. Electrophysiological and pharmacological assays rule out the involvement of voltage-gated Na^+^ channels or constitutively active epithelial-type Na^+^ channels [37, 38]. A three-dimensional model that describes the circulation current and electrochemical profile in the whole cochlea should be constructed with the aim to gain an understanding of the workings of this organ. In vivo, in vitro, and in silico studies will then be necessary to address the mysteries of hearing.

## Robustness and vulnerability in cardiac electrophysiological systems: from the point of view of dynamical system theory (Kunichika Tsumoto, Yasutaka Kurata, Kazuharu Furutani, and Yoshihisa Kurachi)

The heart is a large, hierarchical, complex dynamic system, consisting of cellular, tissue, and organ systems [39]. The accurate and synchronous excitation propagation of action potentials (APs) in cardiomyocytes enables the heart to contract and to function as a blood pump. The persistence of heart contraction during an organism’s lifetime represents the robustness of the system. To the contrary, the heart can also be regarded as a robust system with potential vulnerabilities. Heart diseases resulting from arrhythmias, myocardial infarction, heart failure, among others are typical examples of vulnerabilities in the heart. Such functional failures occur both dynamically and rapidly. In particular, arrhythmias, such as ventricular tachycardia (VT) and ventricular fibrillation (VF), are the major causes of sudden death. The notion to view arrhythmias as dynamical diseases was proposed by physicists in the 1990s [40, 41]. Currently, an understanding of the dynamical aspects of arrhythmias is considered to be of crucial importance in the context of understanding the developmental mechanisms of vulnerability in the heart and how to suppress them. In this section we describe recent advances in our understanding of the dynamical mechanism of early afterdepolarizations (EADs) that are caused in ventricular myocytes, based on in silico studies, and discuss the potential to gain new insights into the prevention and control of EAD-related arrhythmias.

Afterdepolarizations [42], such as EAD and delayed afterdepolarization, which are defined as transient depolarizations during AP phases 2–3 and 4, respectively, are believed to trigger lethal arrhythmias. In particular, EAD is well known to be a repolarization abnormality in the AP and to cause torsade de pointes (TdP) in patients with congenital long QT syndromes (LQTs) [43, 44] and heart failure [45]. In cardiomyocytes, the slow (*I*_Ks_) and rapid components (*I*_Kr_) of the delayed-rectifier K^+^ channel currents play a critical role in the repolarization of APs. Decreases in these repolarization currents prolong the AP duration (APD). Numerous experimental [46, 47] and theoretical studies [48, 49] have shown that excessive APD prolongation results in EADs. These observations imply that the incidence of TdP increases not only in patients with LQTS type 1 (*I*_Ks_ decrease) and type 2 (*I*_Kr_ decrease) and with heart failure, but also in patients treated with drugs that markedly represses repolarization currents [50, 51].

Many experimental studies have suggested that the reactivation of the L-type Ca^2+^ channel current (*I*_CaL_) during AP phases 2–3 in ventricular myocytes is a key mechanism of EAD formation [52, 53]. In silico studies using a mathematical model of guinea pig ventricular myocytes showed that EAD development during AP phases 2–3 is accompanied by *I*_CaL_ reactivation [54, 55]. Also, in our recent theoretical studies [56, 57] using two human ventricular myocyte models developed by Kurata et al. [58] and O’Hara et al. [59], referred to as Kurata and ORd models, respectively, we determined that the occurrence of transient depolarizations during AP plateau resulted from *I*_CaL_ reactivation. An initiation mechanism of the EAD is as follows: under the condition of sufficiently reduced repolarization currents, APDs are markedly prolonged, and the AP repolarization is delayed. The repolarization delay leads to enhancement of the *I*_CaL_ that is transiently increased by *I*_CaL_ reactivation just before the termination of AP repolarization. Increasing reactivation of *I*_CaL_ enhances an inward current component of the net ionic currents (*I*_net_) and causes the inward imbalance of current components in *I*_net_, resulting in a first EAD during AP phases 2–3. Both the ORd and Kurata models [56, 57] as well as the guinea pig model [54] and another human ventricular myocyte model [49] were found to have this initiation mechanism in common.

**Fig. 5:**
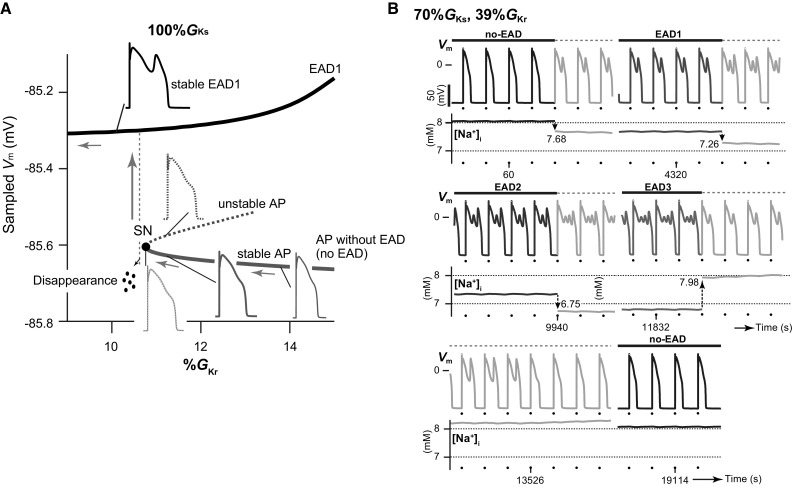
Bifurcation phenomena and tetra-stable action potential (AP) dynamics observed in a human ventricular myocyte model. **a** A one-parameter bifurcation diagram of the sampled membrane potential (*V*_m_) in each AP response as a function of the maximum conductance (%*G*_Kr_) of rapid components (*I*_Kr_) of the delayed-rectifier K^+^ channel currents in the Kurata model with 100%*G*_Ks_. The maximum conductances of the slow component (*I*_Ks_) and *I*_Kr_ of the delayed-rectifier K^+^ channel currents are expressed as a percentage of the control values. The solid and dashed lines represent stable and unstable AP responses, respectively.* EAD* Early afterdepolarization,* SN* saddle-node bifurcation. **b** An example of multi-stable AP dynamics. Simulated tetra-stable AP trains and changes in intracellular Na^+^ concentration ([Na^+^]_i_) observed in the Kurata model at 39%*G*_Kr_ with 70%*G*_Ks_. The arrows (values in mM) indicate each [Na^+^]_i_ perturbation at appropriate times during the simulated AP trains. Colored and gray lines indicate the steady-state and transient responses, respectively. Dots indicate the application of current pulses. Pacing cycle length = 2 s. Each panel was modified based on Tsumoto et al. [57]

The excitation phenomenon of cardiomyocytes is dynamical and nonlinear. The qualitative properties of dynamical behaviors observed in systems with nonlinearities may change when a parameter of the system changes even slightly. In fields of nonlinear dynamics, such property changes are called *bifurcation* [60, 61]. Examples of phenomena that arise via bifurcation include a transition from an equilibrium state to an oscillatory one or from a regular oscillation to a chaotic state. For example, working myocytes within the heart after an acute myocardial infarction suddenly acquire an automaticity through an accumulation of extracellular K^+^ ion, decrease in K^+^ channel conductances, among others. This is a typical example of bifurcation. While it has been possible to gain an understanding of the EAD initiation mechanism using detailed computer simulation, how the transition between an AP without EAD and that with EAD dynamically occurs has remained unclear. Thus, we attempted to elucidate the transition mechanism by investigating changes in the stability of AP responses observed in the Kurata model without and with periodic stimuli [56, 57]. This is known as the bifurcation analysis, which is a mathematical technique that can be used to evaluate changes in the stability of dynamical behaviors in dynamical systems, as well as the parameter dependency of these phenomena [61, 62]. An AP response without EADs showed prolongation of the APD when the *I*_Kr_ was reduced (blue AP traces in Fig. 5a). Further reduction of these repolarizing currents annihilated the prolonged stable AP response together with an unstable AP response (see overlapped blue and red AP traces in Fig. 5a). The reason for these responses was because the occurrence of a bifurcation resulted in the stable and unstable responses coalescing and disappearing. Such a bifurcation is referred to as a saddle-node bifurcation. Because of this annihilation of AP without EADs, the system dynamics led to a transition to the potentially existing branch of AP with an EAD (gray arrow in Fig. 5a) as a new stable steady state; therefore, the emergence of EADs appeared as a sudden occurrence during reduction of the repolarizing current. Although it has been believed that excessive APD prolongation results in EAD via destabilization of the membrane potential, the emergence of EADs in our ventricular myocyte model was not directly linked to destabilization of the membrane potential via APD prolongation. As a secondary effect of our bifurcation analyses, we found that the ORd and Kurata models exhibited multi-stable AP responses (see example in Fig. 5b) [56, 57]. Such responses are also known as multistability, for example, a bistability (birhythmicity) in which both of two distinct steady states (rhythmic responses) appear with the same parameter set, depending on an initial condition [63]. In rabbit ventricular myocytes and the in silico model, Xie et al. [64] also observed intermittent spontaneous switching between AP responses with EADs and those without EADs that is similar to the bi-stable AP behavior seen with hysteresis; these authors demonstrated that variation in the intracellular Na^+^ concentration ([Na^+^]_i_) played important roles in the occurrence of bi-stable dynamics. The results of our in silico study also suggested that the multi-stable AP dynamics resulted from the variability in [Na^+^]_i_, mediated via Na^+^–K^+^ pump and Na^+^/Ca^2+^ exchanger dynamics [57].

**Fig. 6:**
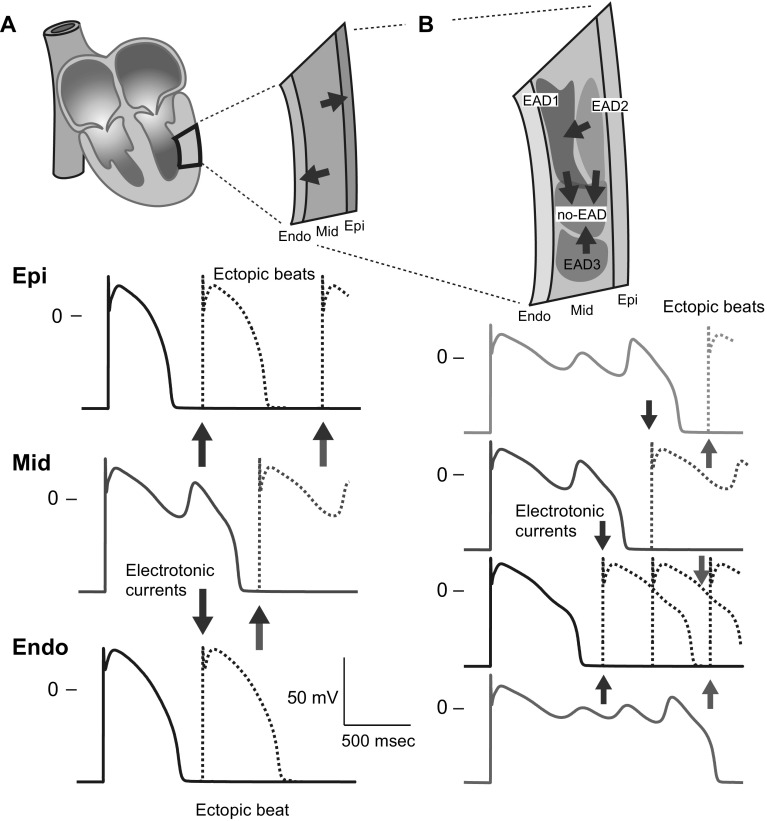
Schematic diagrams illustrating the implication of EAD developments in lethal arrhythmias. **a** Global AP duration (APD) heterogeneity in the ventricular wall (transmural dispersion of repolarizations) and formation of ectopic beats. **b** A possibility of the emergence of local APD heterogeneities (intralayer dispersion of repolarizations in the mid-myocardial layer) by exhibiting multi-stable APs, and an association to arrhythmogenic substrate formation. *Endo* endocardial layer, *Mid* Mid-myocardial layer, *Epi* Epicardial layer

Such multi-stable AP behaviors may have significant implications for EAD-related arrhythmias. Conventionally, a mid-myocardial cell is known to be much more vulnerable to EAD formation than endocardial and epicardial myocytes [50, 65, 66]. Once EADs emerge within the mid-myocardial layer, the marked prolongation of APDs in the mid-myocardial layer globally enhance APD heterogeneities (i.e., transmural dispersion of repolarization) in the ventricular wall (Fig. 6a). This augmentation of APD heterogeneity causes electrotonic interactions (blue arrows in Fig. 2a), i.e., intercellular currents via gap junctions between myocytes, in the boundary zones of the endocardial and mid-myocardial layers and of the mid-myocardial and epicardial layers, leading to re-entrant excitations following ectopic beats. Thus, an arrhythmogenic substrate is dynamically created in the ventricle. In addition, the existence of multi-stable EAD dynamics enables mid-myocardial cells to locally increase the APD heterogeneity (Fig. 6b). This also might be responsible for the generation of re-entrant arrhythmias. State variables, such as ion channel gating states, intracellular ion concentrations, among others are not identical in each mid-myocardial cell. Such state variabilities contribute to the formation of local regions that exhibit each AP response within the mid-myocardial layer (Fig. 6b), causing re-entrant excitations following the regional enhancement of APD heterogeneity (blue arrows in Fig. 6b).

As demonstrated by previous studies [67–69], the relationship between EAD development in single-cell models and arrhythmogenicity in multi-cellular (tissue) models becomes more complicated due to the existence of gap–junction coupling, i.e., electrotonic interaction. In well-coupled mid-myocardial cells in the intact heart, the local APD heterogeneity may decrease because of an averaging effect resulting from the electrotonic effect [69, 70]. However, diminished gap–junction coupling, such as by ageing-related fibrosis, facilitates EAD formation [68, 71], creating suitable conditions for re-entry [48] and leading to the generation of lethal arrhythmias [72, 73].

In addition to progress in experimental techniques, computer simulations will be indispensable techniques to understand complex dynamical behaviors, such as cardiac arrhythmias, and to elucidate their mechanisms. Thus, dynamical system theory may enable us to provide new insights into biological system dynamics.

## Multilevel systems medicine and software platform (Yoshiyuki Asai, Takeshi Abe, and Hiroaki Kitano)

Until the beginning of the present century, in medicine, difficult problems in physiology and life sciences, i.e., complex phenomena, such as pathology, were approached using an elemental reductionism method, which split problems into smaller elements, such as cells, proteins (molecules), etc. As a result, an enormous amount of knowledge was accumulated on each level of a biological system, namely, proteins, cells, organs, and tissues. Systems biology can be described as a paradigm shift toward integral life sciences, through a synthesis of all of this accumulated knowledge into an understanding of life as a multi-level system. Systems biology has also driven the field of ‘omics research,’ which developed at the same time and involves consideration of the entirety of physiology under the moniker “the physiome” [74, 75].

In response to conventional biology, which until now has focused on DNA, proteins, and other substances (“tangibles”) that form biological functions, systems biology research focuses mainly on the dynamics (“intangibles”) of the networks that comprise the tangibles. Therefore, this paradigm shift can also be described as a paradigm shift from the science of “tangibles” to the science of “intangibles”. When biological functions are re-appraised from this perspective, states of illness can be interpreted as disruptions in organismal dynamics that can have deleterious effects on an organism’s survival [76]. Even if individual elements are functioning properly, if the system parameter values, such as reaction coefficients, shift for any reason, the result is a disruption in system dynamics, possibly resulting in a state we recognize as illness. Mackey et al. dubbed this as “Dynamical Disease” [77].

Applying this viewpoint to medical research can allow new therapeutic approaches to be considered [78]. To date, the general approach to illness has involved identification of the factor causing the disease and restoration of the diseased part to its original state. However, under a dynamics-based approach, it is possible that an adjustment of elements other than the element causing the disease could return disrupted dynamics to their original state, thereby enabling therapy. Kitano discusses this point of view from the perspective of the robustness and fragility of living systems, thereby developing the fundamental theory of systems biology [79, 80].

In many cases, a common approach in systems biology research is to conduct simulations using sensitive mathematical models created on the basis of previously accumulated data. Developments in molecular biology and bioinformatics have helped explain the molecular basis of physiological functions and have produced mathematical models at the molecular level, which is one factor driving the recent expansion in systems biology. The results of genome analysis and epigenome analysis from next-generation sequencers have enabled progress, including pathway analysis and estimation of genetic regulation networks [81].

By conducting simulations, we can extract and observed specific dynamics of physiological systems. Empirical data can be regarded as one-sample path static data, even if they are time-series data recorded at specific time points. However, there have been research attempts to understand and intervene in biological functions, specifically changes in the status of systems over time, which are dynamic phenomena. Systems biology can be described as a technology that uses the processes discussed above to bridge the gap between static data and dynamic functions. Because mathematical models are built based on established knowledge of functional networks, they can be thought of as mathematical expressions of collections of knowledge, or collections of hypotheses. The process of creating new knowledge through simulations can be considered to be a process of broadening and deepening existing knowledge.

**Fig. 7:**
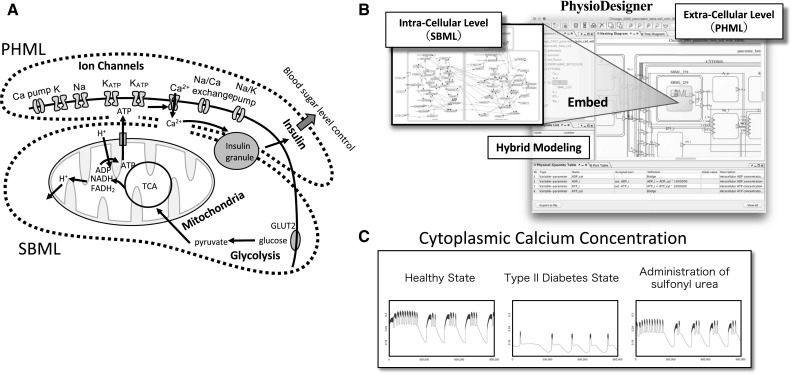
Snapshots of the software PhysioDesigner and the database site. Upper left panel shows Physiome.jp where the model database is opened. Upper right snapshot shows the main window of PhysioDesigner. Lower right and left snapshots are of a simulator Flint, and three-dimension + time domain visualizer, PhysioVisualizer, respectively. *PHML* Physiological Hierarchy Markup Language,* SBML* Systems Biology Markup Language

In tandem with the accumulation of experimental data and developments in computing in the field of systems biology, mathematical models of physiological functions have grown larger, more detailed, and more complex. As such, systematic support from software has become necessary for efficient model development. Efforts are being made to standardize the language in which models are described, with the Systems Biology Markup Language (SBML) [82], CellML language [83], and others having been proposed as candidates. Many tools to support these languages are also being developed. We have proposed a model descriptive language called Physiological Hierarchy Markup Language (PHML) [84] (http://physiodesigner.org/phml/) and have released companion software, PhysioDesigner, which uses an intuitive graphical interface to support multi-level model creation, and a companion simulator, Flint [78]. In addition, a database of models described in PHML has been set up within Physiome.jp [79] (http://physiome.jp). These technologies promote the sharing and re-use of models and data to enable accelerated model development. Snapshots of the software are shown in Fig. 7.

**Fig. 8:**
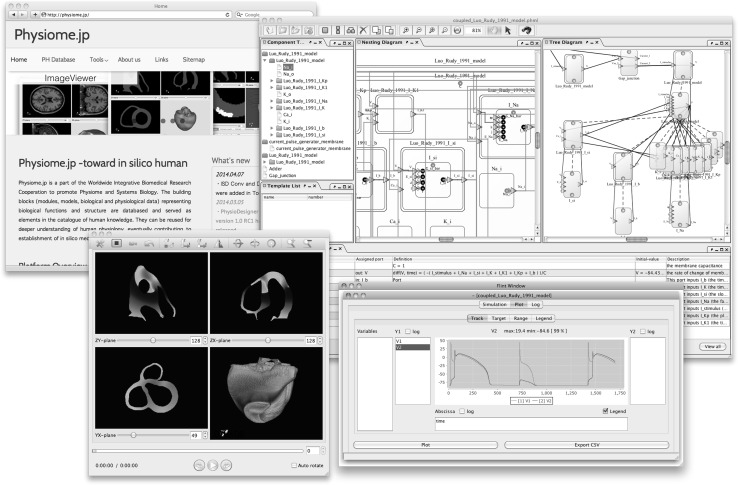
Systems biology approach to pancreatic beta cells. **a** A model diagram of the multi-level insulin secretion system in pancreatic beta cells. **b** A model of pancreatic beta cells created with PhysioDesigner. **c** Simulation examples of calcium dynamics in a cell

Figure 8 shows the time series results of a simulation run in Flint with a model of pancreatic beta cells developed on PhysioDesigner. This model includes each type of ion channel found on the cell membrane, along with the internal cellular processes discussed in the following text [80, 85] (Fig. 8a). After the sugar concentration outside the cell increases, sugars are transported through the cell membrane and sent to the glycolytic pathway, and the pyruvate produced is fed into the citric acid cycle in the mitochondria. The product of the citric acid cycle, ATP, is released from the mitochondria, thereby deactivating the ATP-sensitive K^+^ channels, which in turn promotes the influx of Ca^2+^. An increase in Ca^2+^ concentration in the cytoplasm triggers the exocytosis of insulin granules. Thus, this model shows multiple levels, including molecular processes within the cell and processes in cell-membrane proteins. Notably, the overall model is written in PHML, but the internal mitochondria and glycolytic pathway processes are written in SBML. Therefore, the model is a hybrid model in which SBML models have been imported into a PHML model. PhysioDesigner enables modeling that draws on the strengths of each modeling language (Fig. 8b) [84].

The far left panel of the simulation results (Fig. 8c) shows the dynamics of calcium concentration in the healthy state. Short-cycle oscillating fluctuations can be observed on the peaks of the long-cycle oscillations. Insulin release occurs when the calcium concentration is high. To model type II diabetes from this state, the one parameter value included in the model is changed to a small value. This parameter represents mitochondrial function; therefore, this change can be interpreted as a diabetic state resulting from decreased mitochondrial function, which causes the changes in intracellular calcium concentration dynamics shown in the center panel. Because calcium concentration does not increase sufficiently, the amount of insulin released decreases. The third model, in which yet another parameter value changes, gives the calcium dynamics shown in the far right panel. The waveforms are slightly different compared with the healthy state, but the calcium concentration increased to nearly the same level. This means that the adjustment of the second parameter had a therapeutic effect. In fact, this second parameter represents the opening probability of the ATP-sensitive K^+^ channels targeted by sulfonyl urea, which is one major drug used to treat type II diabetes. The value was decreased in order to close the channel. This simulation shows one example of a search for therapies based on a dynamical understanding of disease, as discussed in the previous section.

## Electronic supplementary material

Below is the link to the electronic supplementary material.

Supplementary material 1 (PDF 1521 kb)
