# Supplementary material for: Integrative and theoretical research on the architecture of a biological system and its disorder
Source: J Physiol Sci. 2019 Mar 13;69(3):433–51. doi: 10.1007/s12576-019-00667-8 (PMC6456489; doi:10.1007/s12576-019-00667-8)
Supplement: Supplementary file 1 — Supplementary material 1 (PDF 1521 kb) [file 12576_2019_667_MOESM1_ESM.pdf]

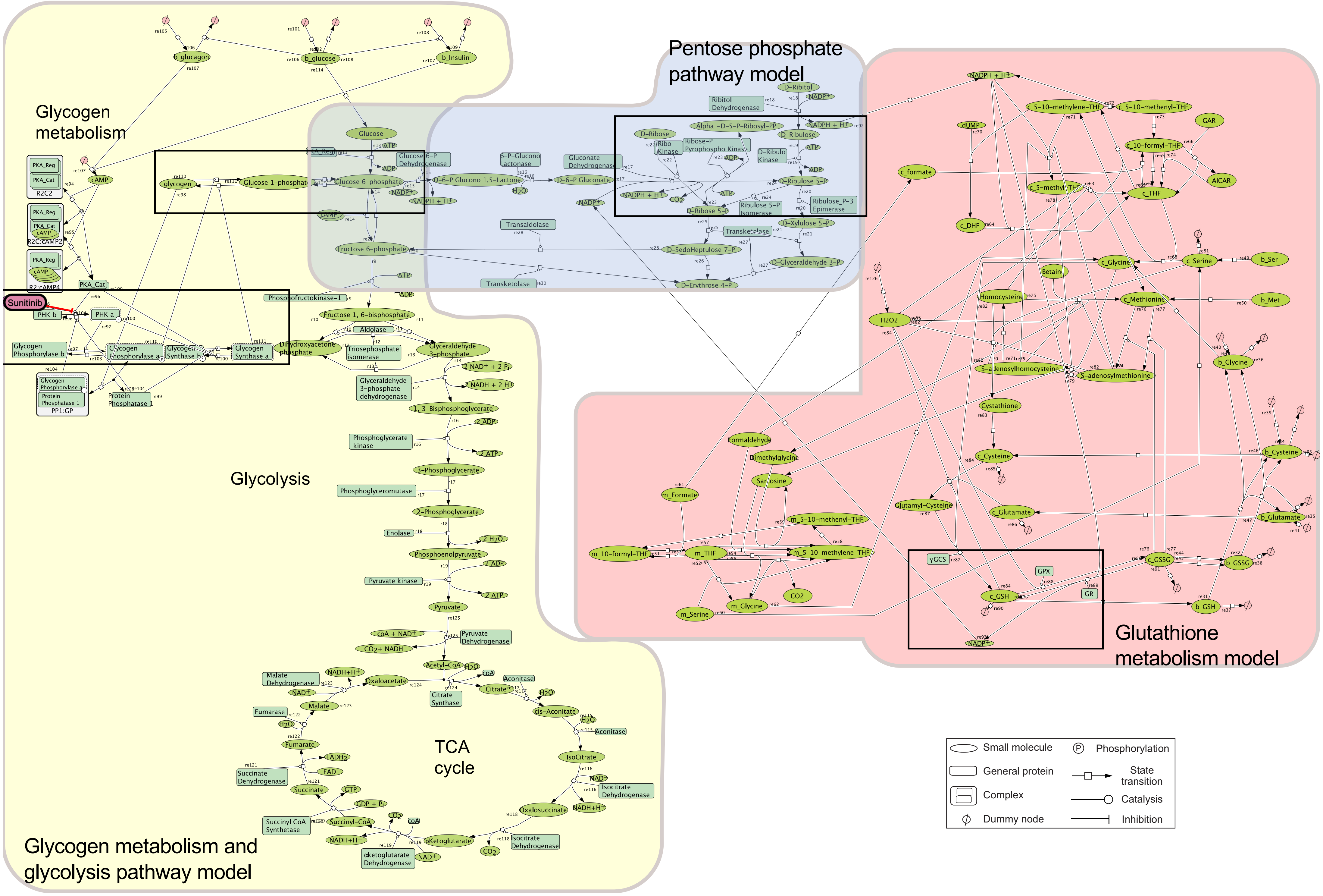

Glycogen metabolism
Pentose phosphate pathway model
Glycolysis
TCA cycle
Glycogen metabolism and glycolysis pathway model
Glutathione metabolism model
Sunitinib
b_glucagon
b_glucose
b_Insulin
Glucose
glycogen
Glucose 1-phosphate
Glucose 6-phosphate
Fructose 6-phosphate
Fructose 1, 6-bisphosphate
Dihydroxyacetone phosphate
Glyceraldehyde 3-phosphate
1, 3-Bisphosphoglycerate
3-Phosphoglycerate
2-Phosphoglycerate
Phosphoenolpyruvate
Pyruvate
Acetyl-CoA
Oxaloacetate
Citrate
cis-Aconitate
IsoCitrate
Oxalosuccinate
oKetoglutarate
Succinyl-CoA
Succinate
Fumarate
Malate
PKA_Reg
PKA_Cat
cAMP
R2C2
R2C:cAMP2
R2:cAMP4
PHK b
PHK a
Glycogen Phosphorylase b
Glycogen Phosphorylase a
Glycogen Synthase b
Glycogen Synthase a
Protein Phosphatase 1
PP1:GP
Phosphofructokinase-1
Aldolase
Triosephosphate isomerase
Glyceraldehyde 3-phosphate dehydrogenase
Phosphoglycerate kinase
Phosphoglyceromutase
Enolase
Pyruvate kinase
Pyruvate Dehydrogenase
Malate Dehydrogenase
Fumarase
Succinate Dehydrogenase
Succinyl CoA Synthetase
oketoglutarate Dehydrogenase
Isocitrate Dehydrogenase
Aconitase
Citrate Synthase
Glucose 6-P Dehydrogenase
6-P-Glucono Lactonase
Gluconate Dehydrogenase
D-6-P Glucono 1,5-Lactone
D-6-P Gluconate
D-Ribitol
Ribitol Dehydrogenase
D-Ribose
Ribo Kinase
Ribose-P Pyrophospho Kinase
Alpha_-D-5-P-Ribosyl-PP
D-Ribulose
D-Ribulo Kinase
D-Ribulose 5-P
D-Ribose 5-P
Ribulose 5-P Isomerase
Ribulose_P-3 Epimerase
D-Xylulose 5-P
Transketolase
Transaldolase
D-SedoHeptulose 7-P
D-Glyceraldehyde 3-P
D-Erythrose 4-P
NADPH + H+
NADP+
c_5-10-methylene-THF
c_5-10-methenyl-THF
c_10-formyl-THF
c_THF
c_5-methyl-THF
c_DHF
dUMP
GAR
AICAR
c_formate
c_Glycine
c_Serine
b_Ser
Betaine
Homocysteine
c_Methionine
b_Met
H2O2
S-adenosylhomocysteine
S-adenosylmethionine
b_Glycine
Cystathione
c_Cysteine
b_Cysteine
Formaldehyde
Dimethylglycine
Sarcosine
m_Formate
m_5-10-methenyl-THF
m_10-formyl-THF
m_THF
m_5-10-methylene-THF
m_Serine
m_Glycine
CO2
Glutamyl-Cysteine
c_Glutamate
b_Glutamate
γGCS
GPX
GR
c_GSH
c_GSSG
b_GSSG
b_GSH
Small molecule
General protein
Complex
Dummy node
Phosphorylation
State transition
Catalysis
Inhibition
